# Correction: Niger's Child Survival Success, Contributing Factors and Challenges to Sustainability: A Retrospective Analysis

**DOI:** 10.1371/journal.pone.0149672

**Published:** 2016-02-12

**Authors:** The PLOS ONE Staff

## Notice of Republication

This article was republished on February 2, 2016, to correct errors in the figure order and to correct author affiliation errors that were introduced during the production process. The publisher apologizes for the errors. Please download this article again to view the correct version. The originally published, uncorrected article and the republished, corrected articles are provided here for reference.

## Supporting Information

S1 File Originally published, uncorrected article. (PDF)

S2 File Republished, corrected article. (PDF)
