# Supplementary material for: Niger’s Child Survival Success, Contributing Factors and Challenges to Sustainability: A Retrospective Analysis
Source: PLoS One. 2016 Jan 19;11(1):e0146945. doi: 10.1371/journal.pone.0146945 (PMC4718615; doi:10.1371/journal.pone.0146945)
Supplement: S1 File — (DOCX) [file pone.0146945.s001.docx]

**Data supplement: Niger’s Child Survival Success: Contributing Factors and Challenges to Sustainability.**

Table of Contents

[A. Additional methods information 2](#_Toc422320190)

[B. Mortality analysis 6](#_Toc422320191)

[C. Additional details regarding the LiST analysis 10](#_Toc422320192)

## Additional methods information

With regards to the indicators including having received two doses of tetanus toxoid during pregnancy, fansidar and early breastfeeding within 1 hour of birth, the MICS questionnaire included all women aged 15-49 with a live birth in the two years preceding the survey, whereas the DHS survey included live births in the five years preceding the survey; the DHS indicators had to be adjusted to reflect the same population group to ensure the best level of comparability across surveys. Furthermore, the indicator relating to the proportion of children under-5, in households who own an insecticide treated net, who slept under a net the night preceding the survey was used. Conventionally, the definition for the proportion of children sleeping under an insecticide treated net does not take into consideration household ownership. When comparing these two indicators across surveys in which both were available, the first indicator, taking into consideration household ownership of nets, often reported higher values.

The Niger Survival and Mortality Survey 2010 is not presented in the LiST analysis in order to maintain comparability across survey indicators though a sensitivity analysis with it included (not presented) yielded similar end year results. The 1998 DHS collected data only for children up to 3 years of age, and as a result, was left out of this analysis for comparability purposes. Coverage data from the SMART surveys were not included in the analysis because the care-seeking variables, a critical component of our analysis, were only included in the 2009 and 2010 SMART surveys and these later surveys excluded some parts of the country.

**Supplementary Table A**: Description of data for mortality and coverage analysis

| Survey and year | Main objectives of the survey | Sampling method | Sample sizes | Sample coverage | Dates of interview | Component of the questionnaire |
| --- | --- | --- | --- | --- | --- | --- |
| DHS | | | | | | |
| 1998 | Estimate socio-economic, demographic and health indicators at national and regional level | Stratified 2-stage cluster sampling; systematic sampling of clusters with PPS; systematic sampling of Households (HH) | 268 clusters, 6007 households, 7863 women 15-49 | National sample | March – July 1998 | HH questionnaire; women 15-49 |
| 2006 | Estimate socio-economic, demographic and health indicators at national and regional levels | Stratified 2-stage cluster sampling; systematic sampling of clusters with PPS; systematic sampling of HH | 342 clusters, 7660 households, 9223 women 15-49 | National sample | January – May 2006 | HH questionnaire; women 15-49 |
| 2012 | Estimate socio-economic, demographic and health indicators at national and regional level | Stratified 2-stage cluster sampling; systematic sampling of clusters with PPS; systematic sampling of HH | 480 clusters, 10750 households, 11,160 women 15-49 | National sample | February – June 2012 | HH questionnaire; women 15-49 |
| MICS | | | | | | |
| 2000 | Estimate socio-economic, demographic and health indicators at national, regional and residential (urban & rural) levels | Stratified 2-stage cluster sampling; systematic sampling of clusters; systematic sampling of HH | 198 clusters, 4321 households, 5664 women 15-49 | National sample | April – August 2000 | HH questionnaire; women 15-49; children under 5 |
| Survival and mortality survey | | | | | | |
| 2010 | Estimate health indicator coverage among children under-5 | Stratified 2-stage cluster sampling; 14 Strata; systematic sampling of clusters with PPS; systematic sampling of HH | 782 clusters, 25,024 households | Sample excludes non-urban areas of Agadez region and nomadic populations | July -September 2010 | HH questionnaire; Coverage and mortality modules |

Box B describes the data sources for the contextual analysis which were used to develop the policy and implementation timeline (figure 10 in the paper).

**Supplementary Box B:** Data sources for the contextual analysis

| Product type | Process of analysis / assessment |
| --- | --- |
| *Documents (policies, programme reports, research reports and papers)* | |
| - Policy and strategic plan documents of the Ministry of Health (National Child Survival Strategy, Poverty Reduction Strategy, Health Development Plan, Strategic Plan for Malaria, National strategy of community based integrated interventions for health) - Catalytic Initiative Support to Integrated Health Systems Strengthening annual progress reports prepared by UNICEF for the Department of Foreign Affairs, Trade and Development (DFATD) Canada. - Programme and research reports from implementation partners and research institutions: UNICEF, USAID, WHO, MSF, LASDEL) - Peer reviewed articles on child survival in Niger | - Review of policies and extraction of information on pivotal events and policy changes related to child survival - Review and extraction of key implementation milestones - Review of documents for information on scope of activities of partners, geographic areas covered, periods of implementation - Pubmed search of literature on child survival in Niger since 2000. Review of articles and extraction of information. |
| *Extraction of socio-political data from databases* | |
| - Extraction of Macro-economic indicators (GDP, expenditure on health) - Extraction of health indicators known to impact on child survival (fertility rate) | - Data extracted from World Bank Niger country dataset and verified against DHS surveys for data collected from that source. |
| *Key informant interviews during country visit* |  |
| - Individual interviews and focus group discussions with Ministry of Health, United Nations staff (WHO/UNICEF) and development partners using a semi-structured interview tool in April 2013. | - 35 interviews conducted, tape recorded and transcribed - Transcripts read by two members of the country evaluation team and common themes related to policy change, partner alignment and collaboration and programme implementation were identified. |

## Mortality analysis

Method of computation of under-five mortality rates

Under-five mortality rates were computed for successive two year and single year periods preceding the 2012 DHS using direct methods based on complete birth histories as recommended by the DHS statistical guide [1]. For each five-year period, age-specific mortality probabilities were computed for eight age groups: 0, 1-2, 3-5, 6-11, 12-23, 24-35, 36-47, 48-59 month using a synthetic cohort concept. This approach involves approximations of the number of children who enter an age range during a specific period. The resulting survival probabilities of death for each age interval were chained together to estimate the survival probability at age five, which was then converted into the under-five mortality rate.

Due to smaller sample sizes, traditionally 5-year time periods for mortality analysis have been used  since shorter periods often result in large sampling errors because of small sample sizes. However, in recent times with increasingly large samples included in the DHS surveys, using shorter time periods are more viable. The benefits of shorter time periods of analysis allow for the analysis to measure the effects of particular events, such as a drought, or war, and allow for significant changes in the pace of change of child mortality to be discovered earlier. This analysis reported on two-year and single year estimates for this purpose. The two-year time periods were used when reporting on mortality trends and average annual rates of reduction as they have less sampling error and narrower confidence intervals.

The death rates in this paper are calculated using the following formula:

The estimated probability of death in age-interval *I in period j*:

**Pr(j*i*) =** $\frac{\boldsymbol{dj}\boldsymbol{i}}{\boldsymbol{nji}}$

where *dj_i_* is the total number of deaths in period j for age-group *i* in 0, 1-2, 3-5, 6-11, 12-23, 24-35, 36-47, 48-59 months and *nj_i_* is the total number of at risk.

The Life Table method then calculates survival probability as the product of 1 minus the conditional probability of death of all age-intervals as follows:

***S(ji)* =** $\prod_{\boldsymbol{i=0}}^{\boldsymbol{i=48-60}} \mathbf{(1-Pr}\left( \boldsymbol{ji} \right)\boldsymbol{)}$for all *i* in 0, 1-2, 3-5, 6-11, 12-23, 24-35, 36-47, 48-59 months.

Therefore Under 5 mortality rate = **(1- *Sj(i)*) x 1000**

The standard errors for the computed under-five mortality were obtained using Jackknife repeated replications procedures [2].

Calculation of average annual change (AAC) in mortality

The Average Annual Change in mortality was calculated using the two-year point estimates for the period between 1989-1990 to 2011 to 2012, to assess how the rate of mortality changed over time.

$${\boldsymbol{r=}\left( \boldsymbol{ln}\left( \frac{\boldsymbol{m}_{\boldsymbol{2011.5}}}{\boldsymbol{m}_{\boldsymbol{1989.5}}} \right) \right)}/\boldsymbol{(2011.5-1989.5)}$$

Tables C and D below give under-5 mortality estimates for two year and single year periods preceding the 2012 DHS with 95% confidence intervals. Data is from the Niger 2012 DHS.

**Supplementary Table C. Two-year under-5 mortality rates from 1989 to 2012 and associated 95% confidence intervals**.

|  |  | **95% CI** | |
| --- | --- | --- | --- |
| **Two-year period** | **Under-5 mortality rate (per 1000)** | **LL** | **UL** |
| 1989-1990 | 285,7 | 177,2 | 394,2 |
| 1991-1992 | 281,4 | 160,2 | 402,6 |
| 1993-1994 | 277,0 | 154,4 | 399,6 |
| 1995-1996 | 281,5 | 132,8 | 430,2 |
| 1997-1998 | 283,6 | 145,5 | 421,8 |
| 1999-2000 | 237,4 | 133,3 | 341,4 |
| 2001-2002 | 212,1 | 97,9 | 326,3 |
| 2003-2004 | 208,3 | 139,6 | 277,1 |
| 2005-2006 | 170,4 | 113,2 | 227,6 |
| 2007-2008 | 144,3 | 112,3 | 176,3 |
| 2009-2010 | 124,4 | 104,7 | 144,1 |
| 2011-2012 | 128,3 | 101,4 | 155,3 |

| **Supplementary Table D. Single year Neonatal, Under-5 and Infant Mortality Rates from 1990 to 2012 and associated 95% Confidence Intervals** | | | | | | | | | | | | | |
| --- | --- | --- | --- | --- | --- | --- | --- | --- | --- | --- | --- | --- | --- |
|  |  | **Neonatal mortality rate** | 95% CI | |  | **Under-5 mortality rate** | 95% CI | |  | | **Infant mortality rate** | 95% CI | |
| **Year** |  |  | LL | UL |  |  | LL | UL |  | |  | LL | UL |
| **1990** |  | 54,7 | 5,1 | 104,2 |  | 272,4 | 135,0 | 409,7 |  | | 110,2 | 32,9 | 187,5 |
| **1991** |  | 65,6 | 9,6 | 121,5 |  | 296,5 | 144,4 | 448,6 |  | | 115,2 | 64,4 | 166,0 |
| **1992** |  | 37,6 | 7,4 | 67,9 |  | 270,2 | 171,0 | 369,3 |  | | 91,8 | 65,2 | 118,5 |
| **1993** |  | 55,5 | 28,3 | 82,8 |  | 273,2 | 149,3 | 397,1 |  | | 124,6 | 73,1 | 176,1 |
| **1994** |  | 44,2 | 9,2 | 79,2 |  | 280,7 | 144,8 | 416,7 |  | | 116,9 | 29,6 | 204,1 |
| **1995** |  | 33,8 | 13,3 | 54,4 |  | 261,7 | 119,6 | 403,7 |  | | 84,5 | 32,3 | 136,7 |
| **1996** |  | 63,3 | 26,7 | 99,9 |  | 300,5 | 143,1 | 458,0 |  | | 118,2 | 62,8 | 173,6 |
| **1997** |  | 52,1 | 31,0 | 73,3 |  | 298,9 | 163,8 | 434,0 |  | | 110,2 | 63,9 | 156,6 |
| **1998** |  | 44,7 | -14,7 | 104,0 |  | 270,0 | 128,7 | 411,3 |  | | 109,1 | 22,4 | 195,8 |
| **1999** |  | 40,9 | 28,1 | 53,7 |  | 245,4 | 141,5 | 349,3 |  | | 100,9 | 50,4 | 151,5 |
| **2000** |  | 33,2 | -0,4 | 66,8 |  | 230,6 | 124,9 | 336,4 |  | | 83,8 | 35,2 | 132,4 |
| **2001** |  | 43,5 | 23,1 | 64,0 |  | 223,4 | 97,2 | 349,7 |  | | 91,5 | 19,7 | 163,3 |
| **2002** |  | 32,4 | -3,9 | 68,8 |  | 202,7 | 104,2 | 301,3 |  | | 81,3 | 38,5 | 124,1 |
| **2003** |  | 37,7 | 6,7 | 68,8 |  | 207,7 | 124,5 | 290,8 | |  | 81,4 | 46,5 | 116,4 |
| **2004** |  | 43,8 | 21,1 | 66,6 |  | 209,3 | 148,7 | 269,9 | |  | 91,5 | 69,8 | 113,2 |
| **2005** |  | 37,6 | 16,7 | 58,6 |  | 177,2 | 105,6 | 248,9 | |  | 75,1 | 33,4 | 116,8 |
| **2006** |  | 35,9 | 22,9 | 48,8 |  | 164,4 | 119,3 | 209,4 | |  | 75,6 | 42,1 | 109,0 |
| **2007** |  | 39,4 | 13,7 | 65,0 |  | 154,9 | 109,6 | 200,2 | |  | 73,6 | 37,0 | 110,1 |
| **2008** |  | 23,4 | 13,5 | 33,3 |  | 131,6 | 110,3 | 152,9 | |  | 53,4 | 39,0 | 67,7 |
| **2009** |  | 28,6 | 17,7 | 39,4 |  | 129,9 | 108,5 | 151,3 | |  | 54,1 | 41,9 | 66,4 |
| **2010** |  | 23,4 | 10,7 | 36,2 |  | 118,9 | 97,0 | 140,7 | |  | 45,2 | 28,5 | 61,8 |
| **2011** |  | 19,8 | 2,8 | 36,8 |  | 113,0 | 85,1 | 140,9 | |  | 42,9 | 12,5 | 73,2 |
| **2012** |  | 24,8 | 5,9 | 43,7 |  | 142,8 | 103,6 | 182,1 | |  | 57,8 | 40,3 | 75,2 |

## Additional details regarding the LiST analysis

Table E shows the data sources used for the baseline characteristics - population, fertility, mortality rates, causes of death and nutrition – for the Niger LiST analyses. Coverage data for key indicators, as well as broader health system indicators, were extracted from all of the available household surveys and other datasets for each time point available and entered into the projections. Data was interpolated linearly between surveys. Table F provides coverage inputs and data sources used in the LiST analysis.

**Supplementary Table E:** Additional data used to create LiST projections

| **Indicator** | **Source** |
| --- | --- |
| Population served | UN estimates [3] |
| Total fertility rate | Demographic and Health Survey 2006, 2012 |
| Stunting, wasting rates | Demographic and Health Survey 2006, 2012 |
| Cause of death | UN estimates [4] |
| Mortality (under-5, infant, neonatal) | UN estimates [3] |

**Supplementary Table F:** Coverage data (%) input and data sources used for LiST analysis

|  | **2006** | **2012** | **Data source** |
| --- | --- | --- | --- |
| Pregnancy |  |  |  |
| Antenatal care (4+ visits) | 14.9 | 32.8 | DHS |
| Tetanus toxoid vaccination (protected at birth) | 23.0 | 50.0 | DHS |
| IPTp for malaria prevention | 0.0 | 59.0 | DHS |
| PMTCT |  |  | UNAIDS |
| Dual ARV | 2.6 | 0.0 |  |
| Option A | 0.0 | 61.3 |  |
| Option B | 0.0 | 23.6 |  |
| Childbirth |  |  |  |
| Skilled birth attendance | 18.0 | 30.0 | DHS |
| Facility birth | 18.0 | 30.0 | DHS |
| *Interventions within the childbirth package:* | 11.4 | 22.4 | LiST default calculations based on facility birth coverage |
| *Clean birth practices* | 9.7 | 20.1 |  |
| *Immediate assessment and stimulation* | 18.0 | 30.0 |  |
| *Labor and delivery management* | 1.3 | 6.0 |  |
| *Neonatal resuscitation* | 5.0 | 16.4 |  |
| *Antenatal corticosteroids for preterm labor* | 5.0 | 16.4 |  |
| *Antibiotics for pPRoM* | 5.0 | 16.4 |  |
| *MgSO4 management of eclampsia* | 5.0 | 16.4 |  |
| *Active management of the third stage of labor* | 0.4 | 1.2 |  |
| *Induction of labor for pregnancies lasting 41+ weeks* | 11.4 | 22.4 |  |
| Breastfeeding |  |  |  |
| Exclusive breastfeeding prevalence <1 month | 12.6 | 35.0 | DHS |
| Exclusive breastfeeding prevalence 1-5 months | 13.4 | 21.0 | DHS |
| Any breastfeeding 6-11 months | 93.0 | 93.0 | DHS |
| Any breastfeeding 12-23 months | 78.7 | 69.9 | DHS |
| Preventive |  |  |  |
| Preventive postnatal care | 12.0 | 17.0 | DHS |
| Complementary feeding (6-9 months) | 54.7 | 58.3 | DHS |
| Vitamin A supplementation | 69.6 | 59.4 | DHS |
| Improved water source | 41.0 | 67.0 | DHS |
| Water connection in the home | 7.0 | 8.1 | DHS |
| Improved sanitation - Utilization of latrines or toilets | 8.8 | 9.6 | DHS |
| Hygienic disposal of children's stools | 14.0 | 22.0 | DHS |
| Ownership of insecticide treated nets (ITN/LLIN) | 43.0 | 61.0 | DHS |
| Vaccines |  |  |  |
| BCG | 64.0 | 84.0 | DHS |
| Polio | 55.0 | 75.0 | DHS |
| DPT | 39.0 | 68.5 | DHS |
| H. influenzae b | 0.0 | 68.5 | DHS (2012 only) |
| HepB | 0.0 | 68.5 | DHS (2012 only) |
| Pneumococcal | 0.0 | 0.0 | LiST default |
| Rotavirus | 0.0 | 0.0 | LiST default |
| Measles | 47.0 | 68.7 | DHS |
| Curative |  |  |  |
| Case management of severe neonatal infection | 8.7 | 8.7 | LiST default calculations based on facility birth coverage |
| ORS - oral rehydration solution | 18.0 | 44.0 | DHS |
| Care-seeking for pneumonia in children | 47.0 | 53.0 | DHS |
| Antimalarials - Artemesinin compounds for malaria | 0.0 | 15.3 | DHS |
| ART | 4.5 | 32.9 | UNAIDS |

* Niger household surveys considered included DHS 1998, 2006 and 2012

Table G below provides estimated deaths averted in Niger between 2006 and 2012

|  | 2006 | 2007 | 2008 | 2009 | 2010 | 2011 | 2012 |
| --- | --- | --- | --- | --- | --- | --- | --- |
| Total under-five deaths | 104000 | 106000 | 108000 | 109000 | 109000 | 108000 | 103000 |
| Additional under-five deaths prevented | 0 | 3000 | 6000 | 9000 | 14000 | 20000 | 26000 |
| Proportion of deaths averted | 0% | 3% | 5% | 8% | 12% | 16% | 20% |
| Predicted under-five mortality rate | 162 | 158 | 154 | 150 | 144 | 138 | 131 |

**Supplementary Table G: Estimated deaths averted in Niger 2006-2012**

Table H below compares the LiST projections between the analysis conducted by Amouzou et al. [5] and this LiST analysis using recently available data including the 2012 DHS

**Supplementary Table H. Comparison of LiST projections between Amouzou et al. [5] analysis and new analysis**

| **Intervention** | **Proportion of deaths averted 1998-2009, Amouzou et al analysis** | **Proportion of deaths averted 2006-2009, new analysis** |
| --- | --- | --- |
| ITN ownership | 25% | 9% |
| Reduction in stunting | 10% | 30% |
| Reduction in wasting | 9% | 0% (4,700 additional deaths in 2009) |
| Care-seeking for malaria | 9% | 9% (ACTs for malaria) |
| Vitamin A supplementation | 9% | 0% (200 additional deaths in 2009) |
| Care-seeking for pneumonia | 5% | 5% |
| ORS and Zinc | 5% | 17% (ORS alone, not zinc) |
| Measles vaccine | 4% | <1% (measles deaths account for only 1% of all child deaths at baseline in 2007) |
| Changes in breastfeeding practices | 3% | 14% |

**References**

1. Rutstein S, Rojas G. Guide to DHS Statistics. Calverton: ORC Macro, 2006.

2. Pedersen J, Liu J. Child mortality estimation: appropriate time periods for child mortality estimates from full birth histories. PLoS Med. 2012;9(8):e1001289. doi: 10.1371/journal.pmed.1001289. PubMed PMID: 22952435; PubMed Central PMCID: PMC3429388.

3. UN Inter-agency Group for Child Mortality Estimation (IGME). Child Mortaltiy Estimates, CME Info 2014 Available from: [www.childmortality.org](http://www.childmortality.org).

4. Liu L, Oza S, Hogan D, Perin J, Rudan I, Lawn JE, et al. Global, regional, and national causes of child mortality in 2000-13, with projections to inform post-2015 priorities: an updated systematic analysis. Lancet. 2014. Epub 2014/10/05. doi: 10.1016/s0140-6736(14)61698-6. PubMed PMID: 25280870.

5. Amouzou A, Habi O, Bensaid K. Reduction in child mortality in Niger: a Countdown to 2015 country case study. Lancet. 2012. Epub 2012/09/25. doi: 10.1016/S0140-6736(12)61376-2. PubMed PMID: 22999428.
