# Supplementary material for: Correction: Niger's Child Survival Success, Contributing Factors and Challenges to Sustainability: A Retrospective Analysis
Source: PLoS One. 2016 Feb 12;11(2):e0149672. doi: 10.1371/journal.pone.0149672 (PMC4752457; doi:10.1371/journal.pone.0149672)
Supplement: S2 File — (PDF) [file pone.0149672.s002.pdf]

PLOS ONE

RESEARCH ARTICLE

# Niger's Child Survival Success, Contributing Factors and Challenges to Sustainability: A Retrospective Analysis

**Donela Besada[1], Kate Kerber[2], Natalie Leon[1], David Sanders[3,4], Emmanuelle Daviaud[1], Sarah Rohde[1], Jon Rohde[1], Wim van Damme[3,5], Mary Kinney[2], Samuel Manda[6,7], Nicholas P Oliphant[8], Fatima Hachimou[9], Adama Ouedraogo[10], Asma Yaroh Ghali[11], Tanya Doherty[1,3]***

**1** Health Systems Research Unit, South African Medical Research Council, Francie van Zijl Drive, Parow, Cape Town, 7535, South Africa, **2** Saving Newborn Lives/Save the Children, Cape Town, South Africa, **3** School of Public Health, University of the Western Cape, Robert Sobukwe Road, Bellville, 7535, Cape Town, South Africa, **4** School of Child and Adolescent Health, Faculty of Health Sciences, University of Cape Town, Rondebosch, Cape Town, South Africa, **5** Institute of Tropical Medicine, Sint-Rochusstraat 2, 2000, Antwerpen, Belgium, **6** Biostatistics Research Unit, South African Medical Research Council, 1 Soutpansberg Road, Pretoria, 0001, South Africa, **7** School of Mathematics, Statistics and Computer Science, University of Kwazulu-Natal, King George V Ave, Glenwood, Durban, 4041, South Africa, **8** UNICEF Head office 125 Maiden Lane, 11th Floor, New York, NY, 10038, United States of America, **9** UNICEF Niger, 2, rue des Oasis - Quartier Ancien Plateau, Niamey, Niger, **10** UNICEF Benin, 01 BP 2289 Cotonou Boulevard de la CEN-SAD, Cotonou, Benin, **11** Ministry of Health, BP 613, Niamey, Niger

* tanya.doherty@mrc.ac.za

CrossMark
click for updates

OPEN ACCESS

**Citation:** Besada D, Kerber K, Leon N, Sanders D, Daviaud E, Rohde S, et al. (2016) Niger's Child Survival Success, Contributing Factors and Challenges to Sustainability: A Retrospective Analysis. PLoS ONE 11(1): e0146945. doi:10.1371/journal.pone.0146945

**Editor:** Jacobus van Wouwe, TNO, NETHERLANDS

**Received:** July 31, 2015

**Accepted:** December 22, 2015

**Published:** January 19, 2016

**Copyright:** © 2016 Besada et al. This is an open access article distributed under the terms of the Creative Commons Attribution License, which permits unrestricted use, distribution, and reproduction in any medium, provided the original author and source are credited.

**Data Availability Statement:** The 2006 and 2012 DHS datasets are available from DHS following a registration process on the DHS website: http://dhsprogram.com/Where-We-Work/Country-Main.cfm?ctry_id=29&c=Niger&Country=Niger&cn=&r=1. The 2000 MICS dataset is available upon request from UNICEF after registration on the UNICEF website: http://mics.unicef.org/surveys. The 2010 Mortality Survey is available from UNICEF upon request to npoliphant@unicef.org.

**Funding:** This evaluation was supported through Catalytic Initiative funding by the Department of Foreign Affairs, Trade and Development Canada

## Abstract

### Background

Household surveys undertaken in Niger since 1998 have revealed steady declines in under-5 mortality which have placed the country 'on track' to reach the fourth Millennium Development goal (MDG). This paper explores Niger's mortality and health coverage data for children under-5 years of age up to 2012 to describe trends in high impact interventions and the resulting impact on childhood deaths averted. The sustainability of these trends are also considered.

### Methods and Findings

Estimates of child mortality using the 2012 Demographic and Health Survey were developed and maternal and child health coverage indicators were calculated over four time periods. Child survival policies and programmes were documented through a review of documents and key informant interviews. The Lives Saved Tool (LiST) was used to estimate the number of child lives saved and identify which interventions had the largest impact on deaths averted. The national mortality rate in children under-5 decreased from 286 child deaths per 1000 live births (95% confidence interval 177 to 394) in the period 1989–1990 to 128 child deaths per 1000 live births in the period 2011–2012 (101 to 155), corresponding to an annual rate of decline of 3.6%, with significant declines taking place after 1998. Improvements in the coverage of maternal and child health interventions between 2006 and

(DFATD), the South African Medical Research Council and UNICEF (contract number 43126165). TD and DS are supported by the National Research Foundation, South Africa. KK and MK are supported by Save the Children's Saving Newborn Lives program, which is funded by a grant from the Bill & Melinda Gates Foundation. The sponsors of the study had no role in the study design, data collection, data analysis, data interpretation or in the decision to submit the paper for publication. The evaluation team had full access to all study data and had final responsibility for the decision to submit for publication.

**Competing Interests:** Dr Fatima Hachimou, Dr Adama Ouedraogo and Mr Nicholas Oliphant are employed by UNICEF. The findings and conclusions in this manuscript are those of the authors and do not necessarily represent the views of UNICEF. This does not alter the authors' adherence to PLOS ONE policies on sharing data and materials.

2012 include one and four or more antenatal visits, maternal Fansidar and tetanus toxoid vaccination, measles and DPT3 vaccinations, early and exclusive breastfeeding, oral rehydration salts (ORS) and proportion of children sleeping under an insecticide-treated bed net (ITN). Approximately 26,000 deaths of children under-5 were averted in 2012 due to decreases in stunting rates (27%), increases in ORS (14%), the Hib vaccine (14%), and breastfeeding (11%). Increases in wasting and decreases in vitamin A supplementation negated some of those gains. Care seeking at the community level was responsible for an estimated 7,800 additional deaths averted in 2012. A major policy change occurred in 2006 enabling free health care provision for women and children, and in 2008 the establishment of a community health worker programme.

## Conclusion

Increases in access and coverage of care for mothers and children have averted a considerable number of childhood deaths. The 2006 free health care policy and health post expansion were paramount in reducing barriers to care. However the sustainability of this policy and health service provision is precarious in light of persistently high fertility rates, unpredictable GDP growth, a high dependence on donor support and increasing pressures on government funding.

## Introduction

Household surveys undertaken in Niger since 1998 have revealed steady declines in under-5 mortality and according to the 2014 UN Inter-Agency Group for Child Mortality Estimation (IGME) report, Niger has already achieved the target for the fourth Millennium Development goal (MDG), to reduce under-5 mortality by two thirds by 2015 [1]. The reduction in the under-5 mortality rate in the last 10 years has been remarkable against a backdrop of limited improvement in the country's economic and social conditions. According to Amouzou et al. [2], the under-5 mortality rate (U5MR) declined from 226 deaths per 1000 live-births in 1998 to 128 by 2009. Contributing factors have included heavy investment in the health sector including construction of health posts starting in 2000, a free maternal and child health policy since 2006 [2], new therapeutic feeding centers for malnutrition and coordinated outreach campaigns for immunization and insecticide-treated bednets (ITNs).

In 2007, Niger was one of six countries to receive support under the Catalytic Initiative (CI)/Integrated Health Systems Strengthening (IHSS) [3] amounting to US $12 million from the Department of Foreign Affairs, Trade and Development Canada (DFATD) and a further US$12.7 million from UNICEF over six years. Funds were used largely to train community health care workers (known as Agents de Santé Communautaire (ASCs), required to have a minimum of secondary school education, selected by the community, and paid $100 per month through a state grant) in integrated community case management (iCCM) for the treatment of diarrhoea, malaria and acute respiratory infections among children under-5. IHSS funds also supported immunisation activities targeting hard to reach populations, procurement and distribution of ITNs, and improved services for women and newborns during pregnancy and the postnatal period. By 2013, 2560 ASCs had been trained throughout the country, covering a population of approximately 3.15 million children under-5 (corresponding to an approximate ratio of 1 ASC per 1200 children)[4]. Community volunteers, known as 'Relais' provide

household level counselling support for healthy behaviours and promote care seeking [5]. The full package of activities is outlined in Table 1.

Recent estimates from IGME [1] and the Institute of Health Metrics and Information [6] suggest, due to overlapping confidence intervals, a slowing and perhaps even a stagnation in the under-5 mortality rate of decline since 2009. Potential threats to the sustainability of Niger's mortality declines are multi-fold, ranging from individual level behavioural and cultural determinants to the surrounding political and economic climate. Niger continues to report persistently high fertility rates [7] resulting in population growth of 3.3% per year [8]. In addition, national and regional political upheaval has resulted in a massive influx of refugees from Mali [9], Nigeria [10] and Libya [11], further compounding population growth. Niger faces sustained food insecurity due to continued unfavourable terms of resource extraction by foreign companies [12], ongoing reductions in local supplies and increased food prices due to global economic policies and recurrent droughts [13]. Moreover, the agriculture sector generates a large proportion of the country's GDP [14] and is significantly affected by climate fluctuations. This is particularly challenging in light of the fact that approximately 80% of the population is rural [15] and dependent on farming for food access and income generation. Economic growth has been slow and volatile in the past decade, and while annual GDP growth rose to 11% in

**Table 1. Interventions supported by the Catalytic Initiative/IHSS in Niger (2007–2013).**

| | |
|---|---|
| **Integrated Management of Childhood Illnesses** | -Procurement of drugs for malaria, diarrhoea, and pneumonia |
| | -Training of ASCs in iCCM and nurses/clinicians in IMCI (including refresher training for trained staff) |
| | -Supervision and monitoring |
| | -Development and printing of training materials |
| | -Basic availability of medication |
| | - Improved capacity of ASCs for treatment of illnesses through training and supplies of diagnostics and treatment |
| | -Screening for malnutrition |
| **Immunisation** | -Reach Every District approach through integrated interventions (immunisation, Vitamin A supplementation, nutrition screening) including: |
| | -Training of health workers on RED and Expanded Programme on Immunisation |
| | -Support for micro-planning |
| | -Support for mobile teams/outreach activities |
| | -Provision of cold chain equipment |
| | -Organisation of immunisation activities targeting hard to reach populations |
| **Antenatal and Neonatal Care** | -Procurement of drugs provided during ANC: ferrous folic acid, sulfadoxine-pyrimethamin, long lasting impregnated mosquito nets (LLIN), de-worming tablets, and tetanus toxoid vaccines at health facilities |
| | -Training of health workers in revised antenatal consultation guidelines at health facilities and community case management of newborns and emergency obstetric care at health posts |
| | -Provision of newborn kits to ASCs |
| **PMTCT** | -Access to HIV testing and counselling during ANC |
| | -Provision of Nevirapine and AZT at district level |
| **Behaviour Change** | -Training of ASCs and relais on key family practices including: breastfeeding, use of ITN and ORS, hand washing |
| | -Integrated communication involving various media and community leaders |

doi:10.1371/journal.pone.0146945.t001

2012, it declined to 4% in 2013 [16] due to low agricultural production and a slowdown in mining. Furthermore, the health system in Niger is heavily donor dependent, with donors contributing 51% of public health expenditure in 2012 [17]. Insufficient replenishment of the Global Fund in recent years [18], a major supporter of the country's health system, had severe consequences for the availability of essential commodities in the public health system [19]. Despite the introduction of free health care for pregnant women and children and a decrease in private health expenditure (mostly out-of-pocket costs), private sources still fund 56% of the total health expenditure, of which 88% comes from households [20].

Much of the effort made in Niger over the past decade has been geared towards improving coverage and access to quality health care for pregnant mothers and their children. A broader analytical approach to Niger's mortality declines will allow for a reflection of the potential for not only sustaining the country's progress, but continuing to build on it through the development of interventions that take into consideration systematic challenges experienced in Niger.

This paper explores Niger's mortality and health coverage data for children under-5 years of age up to 2012 to describe coverage trends of high impact interventions and the result in terms of childhood deaths averted. The Countdown to 2015 for Maternal, Newborn and Child Survival case study on the reduction in child mortality in Niger [2] published in 2012, used available survey data up to 2010 to explore Niger's under-5 mortality declines. We analysed data from the 2010 Survey of Child Survival and Mortality, a recently conducted 2012 Demographic and Health Survey (DHS), and contextual documentation of policies and programmes to provide essential new data to build on the Countdown study. This paper further considers and discusses the sustainability of these trends in light of Niger's rapidly rising population, and broader economic and political challenges.

# Methods

## Data sources

The evaluation included all nationally representative household survey datasets available: 2000 Multiple Indicator Cluster Survey (MICS) [21], 2006 DHS [22], 2010 Survival and Mortality Survey [23], and 2012 DHS [24]. The 1998 DHS collected data only for children up to 3 years of age, and was therefore excluded from this analysis for comparability purposes, as many of the indicators that were analysed across survey years included children up to 5 years of age. Estimates of intervention coverage at population level from the 2006 and 2012 DHS were used as inputs to the Lives Saved Tool (LiST) to model the overall estimated lives saved as well as the additional independent impact of care seeking at the community level on deaths averted. Birth and death history data of all children of women aged 15 to 49 years sampled in the 2012 DHS was used to calculate under-5 mortality. Full survey datasets with district sampling weights were used for the analysis. For further details on the surveys included in the analysis see Table A in S1 File. Adjustments were made to align indicator definitions across the DHS, MICS and 2010 Survival and Mortality surveys (S1 File: Additional methods information).

Contextual information about child health policies, CI/IHSS implementation and other relevant child health programmes was obtained through a desk review of documents and databases, and key informant interviews conducted during a 10-day country visit (April 2013). The information gathered from these sources was used to compile a policy and programme timeline (Fig 1). For further details on the contextual analysis see Box B in S1 File.

## Statistical analysis

For under-5 mortality estimation, we used a direct method based on the synthetic cohort approach [25, 26]. Age-specific mortality probabilities for narrow age ranges and defined

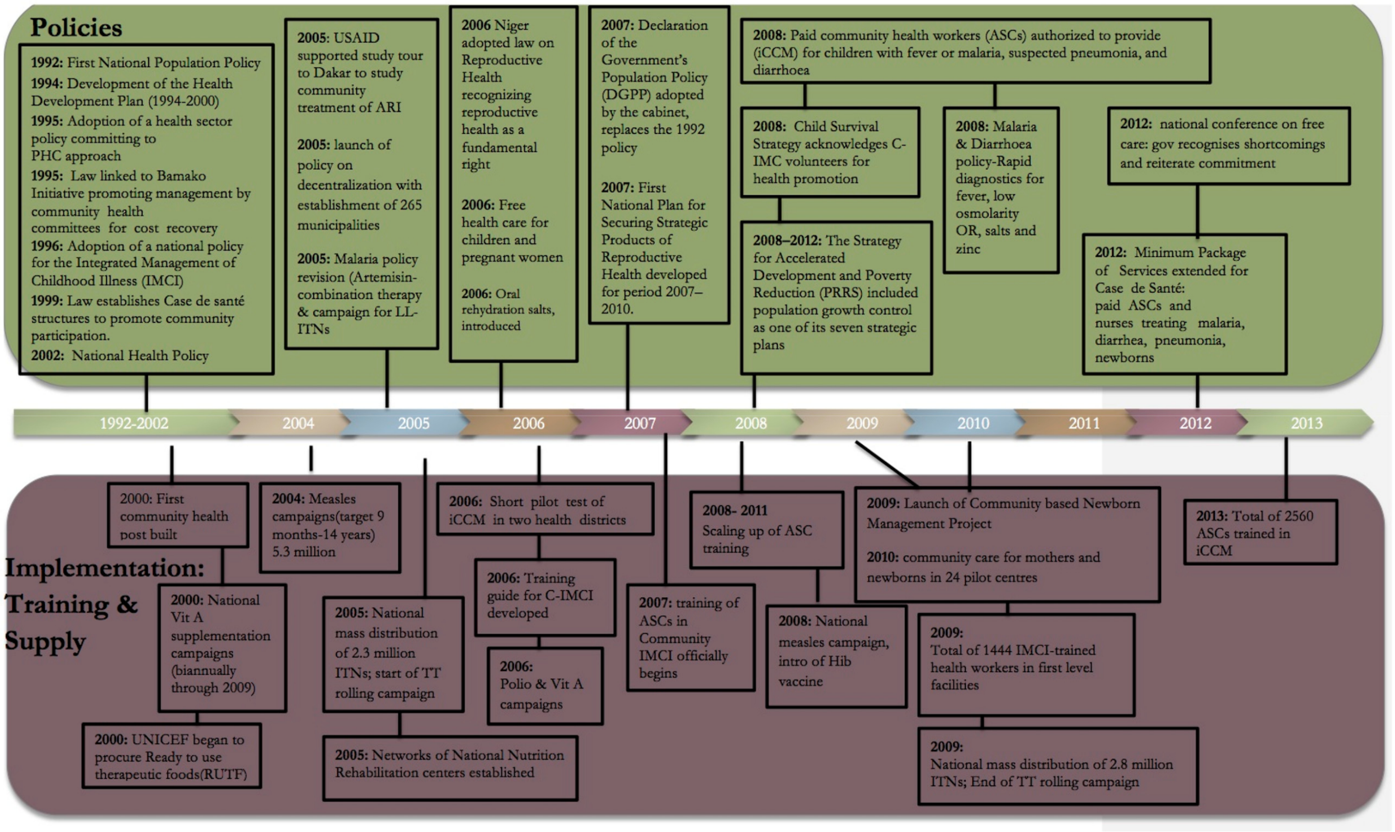


**Fig 1. Major policy changes and activities related to child survival in Niger, 1992–2013.**

doi:10.1371/journal.pone.0146945.g001

periods were calculated using death events and exposures. These probabilities were combined to compute the probability that a child has not died before reaching age 5 years. Two year periods were used beginning with two years before the survey, and survival probabilities were calculated over age ranges; 0, 1–2, 3–5, 6–11, 12–23, 24–35, 36–47, 48–59 months as recommended by DHS (S1 File: Mortality analysis) [26]. The standard errors for the computed mortality estimates were obtained using the Jackknife variance estimation, a repeated sampling method [25]. A series of mortality estimates were obtained by deleting and replacing each primary sampling unit; this produced a sample of under-5 estimates, from which the variance was computed in turn. For more information see see S1 File: Mortality analysis.

All relevant coverage indicators from each survey dataset were calculated using standard definitions for tracking progress towards MDG 4 [27]. Anthropometric indicators including stunting and wasting in children under-5 years of age were calculated from raw survey data using the 2006 WHO child growth standards. For stunting and wasting, moderate and severe forms were aggregated. Significant differences in coverage of pertinent indicators between survey years were determined based on the overlap in the 95% confidence intervals around the estimates.

Changes in care-seeking patterns were also analysed, with a particular focus on community level care-seeking. Data relating to care sought and received for fever, suspected pneumonia and diarrhoea were extracted from available surveys.

The sampling design of these household surveys such as regional and rural/urban stratification, clustering at enumeration areas and sampling weights (due to non-proportional

sampling) were taken into account. Stata (version 12) was used for coverage and mortality trend analyses.

The retrospective LiST analysis investigated the extent to which changes in mortality could be associated with changes in intervention coverage between 2006 and 2012. Annual coverage values were interpolated linearly between the 2006 and 2012 household survey data points, using only DHS to maintain comparability of sources. In this analysis, anthropometric data were entered directly into the model in order to calculate deaths averted due to decreases in stunting and wasting rates. LiST methods and inputs have been widely published [28–30]. Further details on the LiST analysis can be found in S1 File: Additional details regarding the LiST analysis.

To quantify the impact of increased health access through community level services on child mortality, we used LiST to estimate the deaths averted between 2006 and 2012 from all care-seeking for childhood illness at appropriate providers (i.e. not including pharmacies, shops and traditional practitioners), using methods described in detail elsewhere [31]. We compared this with a scenario where care-seeking at community level (i.e. from ASCs and health posts) was removed from the coverage estimates in order to determine the number of lives saved that could be attributed to the introduction of community level services.

This study was approved by the ethics committee of the South African Medical Research Council (EC026-9/2012). Approval was also provided by the UNICEF Niger country office. Data for the analysis of intervention coverage and mortality was taken from secondary sources (nationally representative household surveys) which are anonymized and de-identified prior to public release.

## Results

The national mortality rate in children under-5 decreased from 286 child deaths per 1000 live births (95% confidence interval 177 to 394) in 1989–1990 to 128 child deaths per 1000 live births in the period 2011–2012 (101 to 155), corresponding to an annual rate of decline of 3.6% (Fig 2). Significant declines only began to take place after 1998 however.

Table 2 reports on the coverage changes for select interventions across available survey years. Coverage of both one and four or more antenatal visits rose significantly between 2006 and 2010; coverage of one antenatal visit remained stable thereafter with a slight further increase in four or more antenatal visits between 2010 and 2012. The rise in antenatal care attendance is mirrored by the provision of Fansidar for intermittent preventive treatment of malaria in pregnancy (IPTp) which rose from virtually no coverage in 2006 to 63% in 2012 and the proportion of pregnant women who received two doses of tetanus toxoid vaccine which rose by 27 percentage points between 2006 and 2012. Other improvements included increases in skilled birth attendant at delivery, which doubled between 2000 and 2006, but then remained stable. No significant change was noted in postnatal care received by women who delivered at home in the first two days after delivery (Fig 3).

Less progress has been made in infant and young child feeding indicators (Fig 4). The proportion of children breastfed within one hour of birth and complementary feeding of children aged 6–9 months remained stable between 2006 and 2012. Rates of exclusive breastfeeding rose from virtually 0% to 24% by 2010, with no significant changes thereafter.

Childhood immunisation (Fig 5) saw substantial gains during the period of analysis, with 3 doses of diphtheria, pertussis and tetanus (DPT) vaccine and measles coverage rising between 2000 and 2010 28% to 69% and 36% to 69% respectively, without much change thereafter. Since 2009, the DPT vaccine is delivered as a pentavalent including haemophilus influenza B and pneumococcal vaccines. Vitamin A coverage increased by 11 percentage points between 2000 and 2006 but declined back to 2000 levels by 2012.

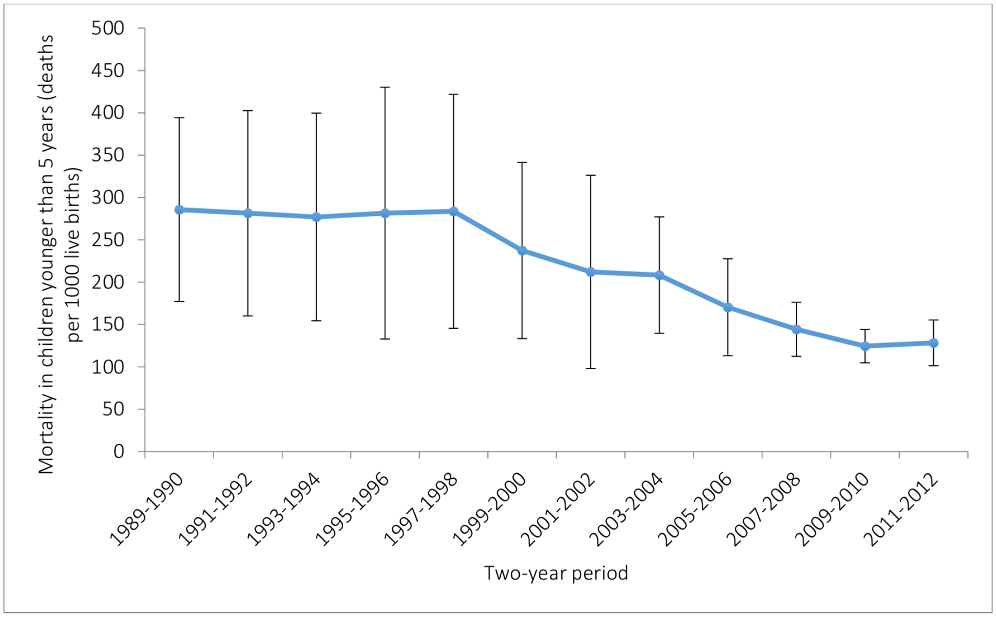


**Fig 2. Under-5 mortality rate in Niger 1989–2012.** Data are from analysis of the 2012 national DHS survey in Niger. Vertical lines show 95% CIs for survival probabilities. Dates on the x-axis represent the 2 year periods preceding the 2012 Niger DHS.

doi:10.1371/journal.pone.0146945.g002

**Table 2. Coverage changes for select interventions including 95% Confidence intervals.**

| Indicator | Niger | | | |
|---|---|---|---|---|
| | MICS 2000 (95%CI) | DHS 2006 (95%CI) | Mortality Survey 2010 (95% CI) | DHS 2012 (95%CI) |
| Tetanus toxoid vaccination of pregnant women (at least 2 doses) | 13(11–15) | 23(21–25) | 48(46–50) | 50(48–52) |
| At least one dose of IPTp | | 0.3(0–0.3) | | 63(60–66) |
| Early breastfeeding within one hour of birth | | 47(43–50) | 44(42–45) | 51(50–56) |
| Exclusive breastfeeding (0–6 months) | 1 (0.5–2.6) | 13(11–16) | 27(25–29) | 22(19–26) |
| Vitamin A supplementation (6–59 months) | 59 (54–64) | 70(67–73) | | 59(56–62) |
| Under 5 sleeping under an ITN | 6(4–12) | 9(8–11) | 79(78–80) | 26(24–29) |
| DPT3 immunisation (12–23 months) | 28(24–33) | 40(35–44) | 68(66–70) | 69(65–72) |
| Measles immunisation (12–23 months) | 36(31–40) | 47(43–52) | 70(68–72) | 69(66–71) |
| Care-seeking of suspected pneumonia | 27(22–33) | 47(42–53) | 54(51–57) | 53(47–60) |
| Care-seeking for fever | 19(16–22) | 45(41–49) | 56(54–58) | 51(47–55) |
| Treatment with any antimalarial for fever | 48(44–52) | 33(30–36) | | 19(16–22) |
| ORS coverage | 14(12–17) | 18(15–20) | 36(34–37) | 44(41–48) |
| Postnatal care | | 12(10–15) | | 17(15–19) |
| Atleast one ANC visit | | 47(44–50) | 83(81–85) | 86(84–87) |
| 4 or more ANC visits | | 15(13–17) | 25(23–28) | 33(31–35) |
| Skilled Birth Attendant | 15(12–19) | 33(30–36) | 33(31–35) | 30(27–32) |
| Complimentary Feeding (6–9 months) | 52(45–59) | 55(49–60) | 41(39–47) | 58(54–62) |

doi:10.1371/journal.pone.0146945.t002

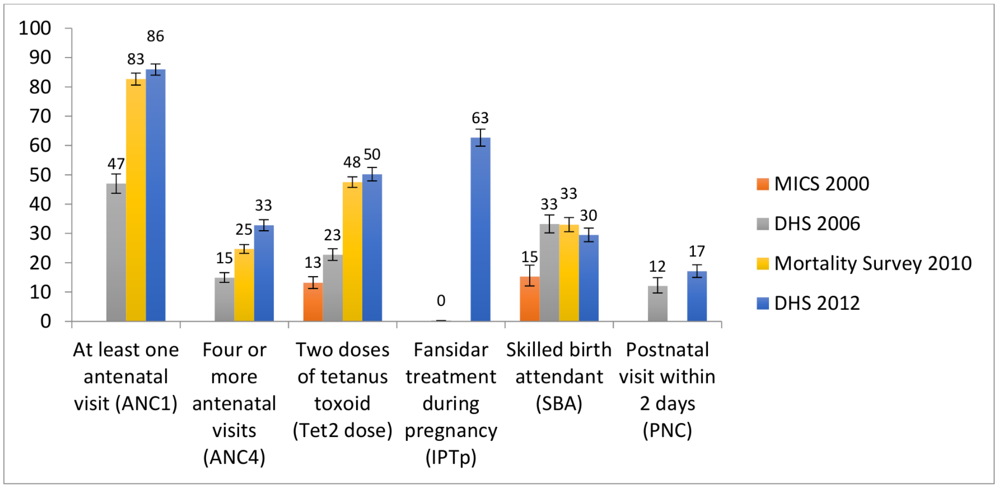


**Fig 3. Coverage of pregnancy related indicators.** Bars represent 95% CIs; two doses of tetanus toxoid for all survey years with the exception of 2010 correspond to children under-2 whereas the 2010 mortality survey only includes children under 1 year; postnatal visit within 2 days is for out of facility deliveries.

doi:10.1371/journal.pone.0146945.g003

Figs 6 and 7 compare levels of wasting and stunting in children under-5 between 2000 and 2012. Wasting rates declined between 2000 and 2006 across all age groups, but rose by 2012 to rates similar to those seen in 2000, with the exception of children aged 36–59 months, whose 2012 wasting rates were double that of 2000 (Fig 6). Stunting rates in Fig 7 follow an opposite trajectory, with non-significant increases between 2000 and 2006. The drops in stunting rates between 2000 and 2012 were significant for all age groups with the exception of children aged 6–11 months.

Some notable increases are seen in other interventions targeting children under-5 between 2006 and 2010 (Fig 8) including the proportion of children sleeping under ITNs and the proportion of children with fever taken to an appropriate provider. Care-seeking for fever remained stable thereafter, with coverage of ITNs dropping to 27% in 2012 from 79% in 2010.

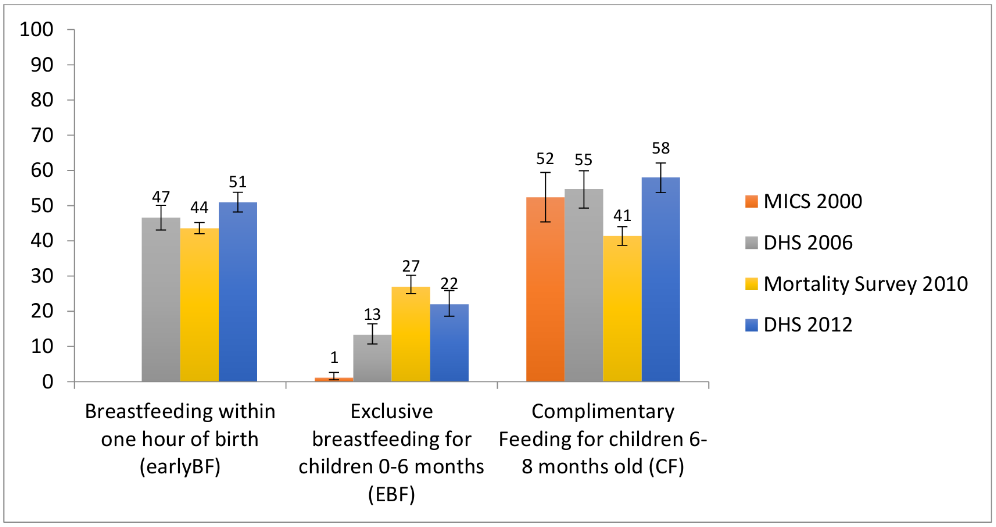


**Fig 4. Infant feeding.**

doi:10.1371/journal.pone.0146945.g004

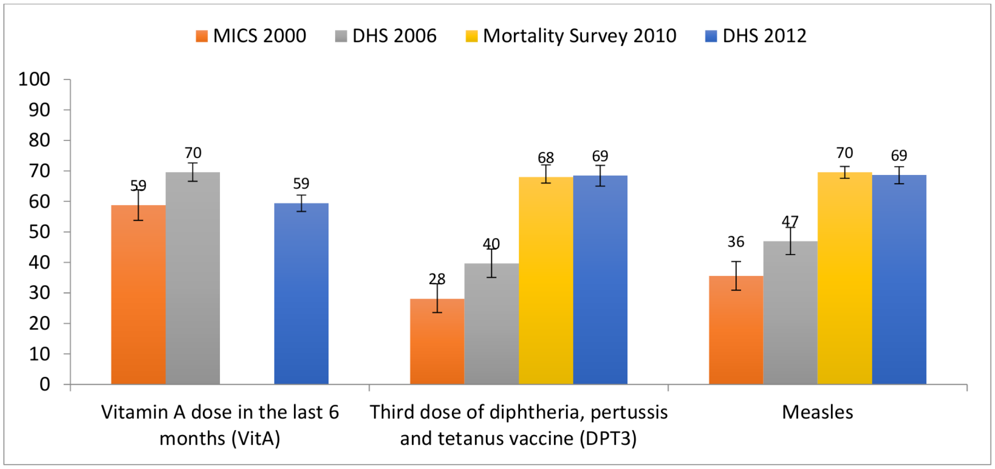


**Fig 5. Childhood immunization.**

doi:10.1371/journal.pone.0146945.g005

Oral rehydration salts (ORS) coverage remained stable between 2000 and 2006, but rose from 18% to 44% by 2012. Coverage of any anti-malarial treatment declined steadily between 2000 and 2012 whilst coverage of artemisinin-based combination therapy (ACTs), introduced in 2005, increased from zero to 15% by 2012. Care-seeking for pneumonia increased significantly only in the period between 2000 and 2006, with no further significant increases thereafter.

Fig 9 depicts changes in overall care-seeking behaviour between 2006 and 2012. There is evidence of an increase in care-seeking at the community/health post level, with overall care-seeking more than tripling from 4% to 15%; increases in community level care seeking for each disease was similar (approximately 10%) (Table 3). Care-seeking also increased significantly at other levels of the public sector (clinics and public hospitals) with coverage increasing from 14% to 31% over the same period, while the proportion of the population who did not seek care at all for any of the three diseases declined from 52% to 42% and the proportion of those

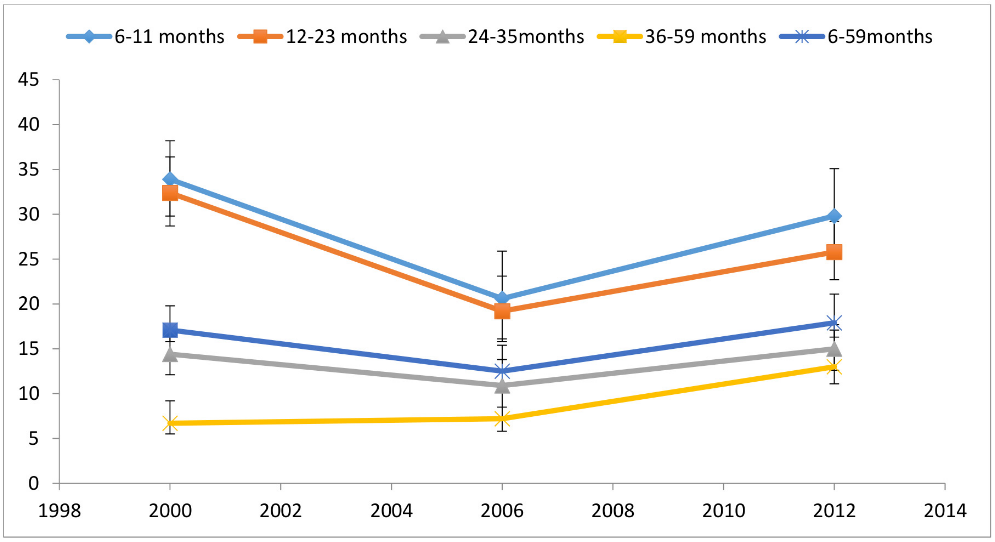


**Fig 6. Prevalence of wasting by age in children in Niger.** Moderate and severe (<-2 z score). Data taken from the 2000 MICS, 2006 DHS and 2012 DHS.

doi:10.1371/journal.pone.0146945.g006

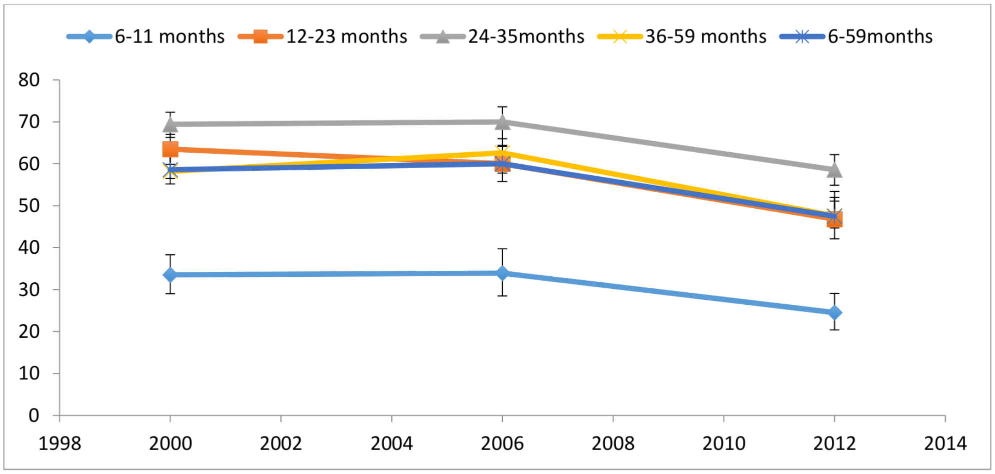


**Fig 7. Prevalence of stunting by age in children in Niger.** Moderate and severe (<-2 z score). Data taken from the 2000 MICS, 2006 DHS and 2012 DHS.

doi:10.1371/journal.pone.0146945.g007

seeking private care dropped to virtually zero. Taking into consideration all appropriate sources of care, care seeking for fever and pneumonia increased by approximately 10 percentage points, although those changes were not statistically significant, while care seeking for diarrhoea increased by over 30 percentage points (Table 3).

In 2012, the estimated main causes of under-5 deaths were malaria (19%), pneumonia (16%), diarrhoea (12%), and preterm complications (9%) [32]. According to these modelled estimates, measles deaths dropped from 5% of all under-5 deaths in 2000 to less than 1% in 2012. Causes of death in the first month of life accounted for 27% of all under-5 deaths in 2012. Using the baseline under-5 mortality rate of 162 in 2006 from IGME [1], the under-5 mortality rate predicted by LiST based on measured coverage change between 2006 and 2012 was 131, compared to the 2012 DHS point estimate of 127 (95% uncertainty interval 119 to 136) and the 2012 IGME estimate of 110 (90–133). Approximately 26,000 deaths of children under-5, including 5,100 neonatal deaths, were averted in 2012 due to changes in coverage: a 20% reduction compared to the 2007 baseline (Table G in S1 File). In 2012, decreases in stunting rates accounted for 27% of all deaths averted. However, increases in wasting rates resulted in an

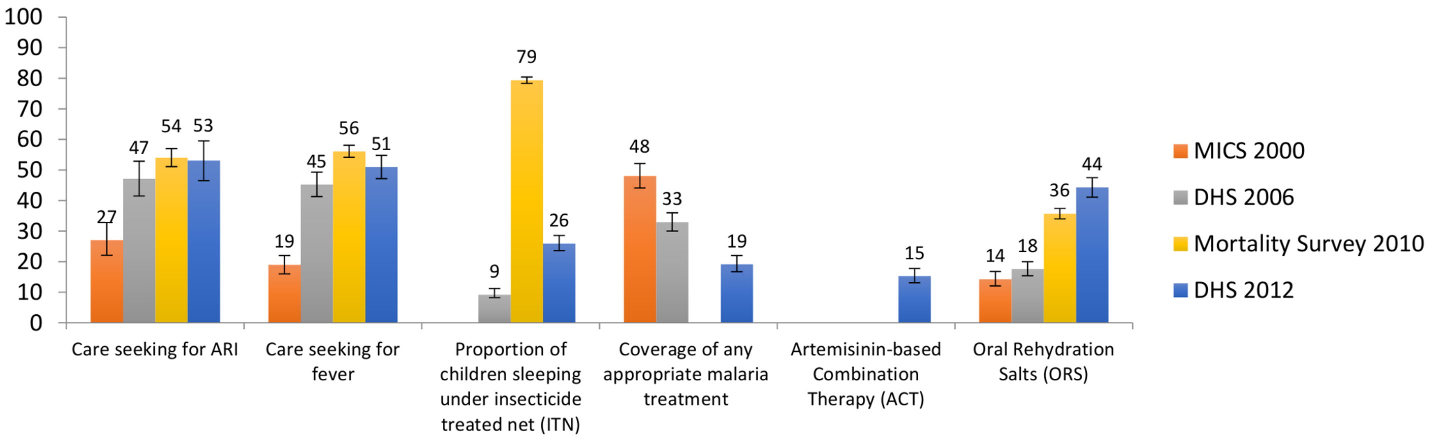


**Fig 8. Coverage of care for childhood illnesses.**

doi:10.1371/journal.pone.0146945.g008

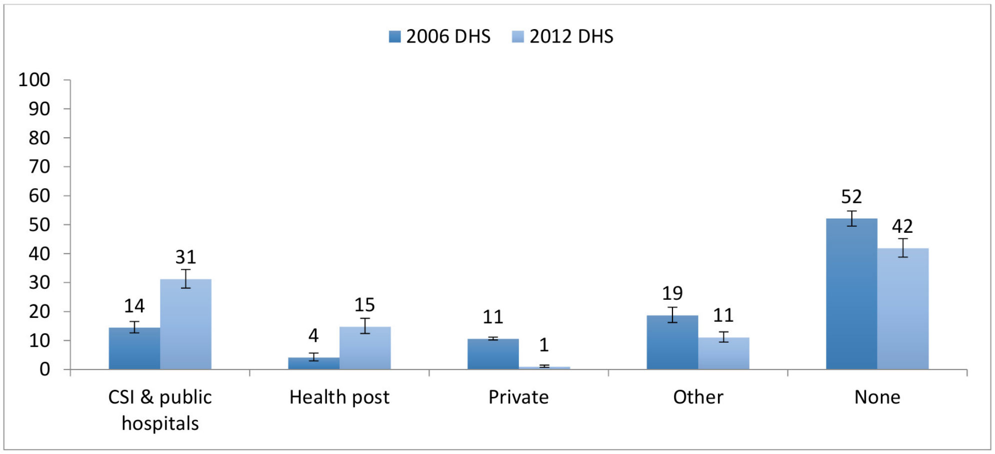


**Fig 9. Care seeking patterns in Niger for diarrhoea, pneumonia and malaria in children under-5 (2006–2012).** CSI = Centres de Santé Integres (Integrated Health Centres).

doi:10.1371/journal.pone.0146945.g009

additional 10,500 deaths in 2012 compared to 2007, nearly the same amount as were averted through stunting decreases. The Hib vaccine introduced in 2009 accounted for 14% of the deaths averted. Vaccines that were already high in coverage in 2006, such as measles, did not show up as contributing to a high proportion of deaths averted because deaths due to measles had declined significantly in recent years. Increases in care-seeking for suspected pneumonia, ACTs for malaria and ORS for diarrhoea resulted in 6200 deaths averted in 2012. Increases in ORS coverage contributed the most amongst curative interventions for childhood illness, accounting for 14% of all deaths averted. Decreases in vitamin A supplementation resulted in an estimated 500 additional deaths (Fig 10).

We compared these results with results reported in the study by Amouzou et al. [2] which used 1998 as the baseline year and the 2010 Survival and Mortality Survey as the endline source of data. While both analyses report substantial declines in under-5 mortality the inclusion of the 2012 DHS presents less optimistic progress. The inclusion of the high impact Hib vaccine results in other interventions having proportionately less impact in our analysis. While ITN

**Table 3. Changes in coverage of care-seeking for 3 childhood illnesses between 2006 and 2012.**

| | All appropriate sources (CSI and public hospitals, health posts, private) | Health posts only |
|---|---|---|
| *Care-seeking for fever* | | |
| 2006 | 45% | 7% |
| 2012 | 53% | 17% |
| *Care-seeking for suspected pneumonia* | | |
| 2006 | 47% | 7% |
| 2012 | 56% | 18% |
| *Care-seeking for diarrhoea* | | |
| 2006 | 18% | 3% |
| 2012 | 50% | 15% |
| Care-seeking for all three diseases | | |
| 2006 | 29% | 4% |
| 2012 | 47% | 15% |

CSI = Centres de Santé Integres (Integrated Health Centres)

doi:10.1371/journal.pone.0146945.t003

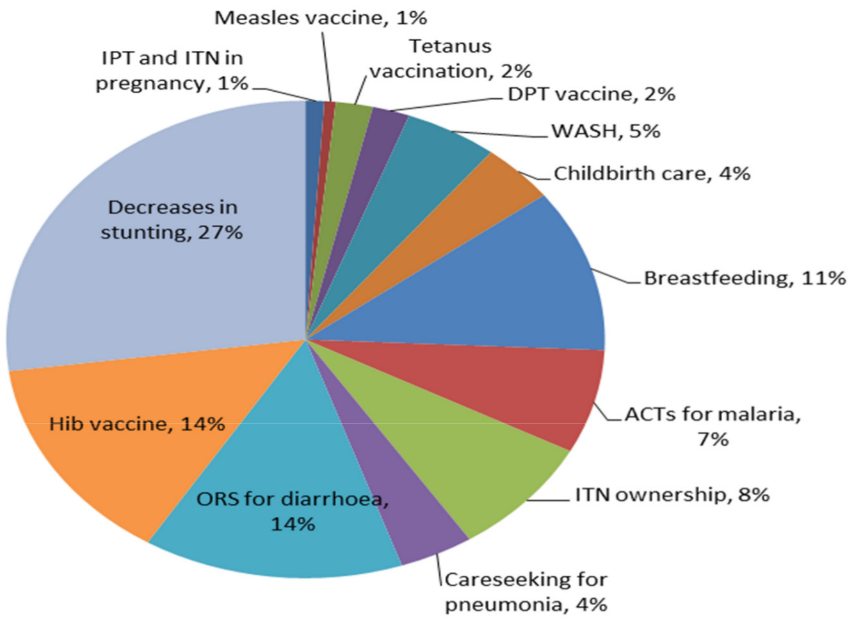


**Fig 10. Proportion of under-5 deaths averted by changes in interventions between 2006 and 2012.**

doi:10.1371/journal.pone.0146945.g010

ownership and ACTs still account for a large proportion of deaths averted, the impact of these interventions is lower given the smaller coverage increases seen in ITNs in the 2012 DHS (Table H in S1 File).

After removing the direct impact of decreases in stunting and wasting, changes in care-seeking for diarrhoea, pneumonia and malaria at health post level were responsible for an estimated 7,800 additional deaths averted in 2012. In this scenario, increases in care-seeking at the health post were responsible for 12% fewer deaths compared to a hypothetical scenario where the health posts and ASCs did not exist and the only available care would be at the facility level. While overall care-seeking for pneumonia and fever increased, care in the private sector declined between 2006 and 2012 resulting in a reduction of deaths averted at these levels and allowing for an even greater impact of the community care-seeking component (Table 4).

Major policy and programme implementation related to maternal and child health took place in Niger from the mid-1990s to 2013 (Fig 1) that help explain the observed changes in

**Table 4. Impact of increased health access through community level services on child mortality.**

| | Estimated child deaths averted in 2012 | |
|---|---|---|
| | **From care seeking from all appropriate places of care*** | **From care seeking from community-level providers alone** |
| Care-seeking for diarrhoea | 6,500 | 2,500 |
| Care-seeking for pneumonia | 2,400 | 2,600 |
| Care-seeking for fever (malaria) | 2,300 | 2,700 |
| Total | 11,200 | 7,800 |

*public health facility, private health facility, health post, community health worker

doi:10.1371/journal.pone.0146945.t004

health intervention coverage and care seeking practices. In the mid-1990s a national policy for the Integrated Management of Childhood Illness (IMCI) was adopted. The first community health posts were built in 2000 to increase health access for communities further than 5km from a health facility, and by 2007 nearly 2000 health posts existed. In 2008, the National Child Survival Strategy was implemented and allowed for ASCs, placed in the posts, to provide integrated community case management (iCCM) to children under-5. Other policies included the provision of free care for pregnant mothers and children in 2006, and successive roll outs of ITN distribution campaigns and outreach activities for immunization.

## Discussion

This study has used the latest available demographic health survey data from Niger to describe coverage changes in high impact health interventions and the resulting childhood mortality trends and considers the sustainability of these trends in light of the broader contextual analysis conducted. The LiST analysis was used to measure the impact of the country's investment in expanded health care access on childhood deaths averted. Niger has achieved considerable success in reducing childhood mortality, attaining nearly double the average rate of reduction of countries in sub-Saharan Africa [1]; this reduction is remarkable in light of the fact that mortality rates were amongst the highest in the world in the 1990s and the country remains amongst the poorest globally [33].

This success is plausibly due to major policy changes and health programme delivery in the country, including the construction of health posts and the provision of free care to pregnant mothers and children, contributing to increased health care access, as well as successive implementation of programmes including the CI/IHSS in 2007 resulting in the training and equipping of ASCs to deliver iCCM. Provision of an antenatal care (ANC) kit in 2006 resulted in an increase in access to such services as bed nets, iron and folic acid, deworming tablets, Sulfadoxine/Pyrimethamine for IPTp and tetanus toxoid vaccines to pregnant women. In 2012, approximately 26,000 childhood deaths were averted due to reduced childhood stunting, rapid scale up of childhood immunisation, most notably the Hib vaccine which accounted for 14% of deaths averted, increases in care seeking for suspected pneumonia and the provision of curative services to children under-5 including ACTs for malaria and ORS for diarrhoea, the last accounting for 14% of deaths averted. Maintaining coverage of interventions already achieving a relatively high level of success, such as measles vaccination, has been essential to progress thus far.

Although malaria services were one of the key focuses of the iCCM programme, the progressive decline in coverage of malaria treatment for fever down from a peak of 48% in 2000 to 19% by 2012 could possibly be attributed to the introduction of Rapid Diagnostic Tests (RDTs) in 2008, increasing the accuracy of diagnosis. However, declining antimalarial coverage preceded the introduction of RDTs by many years and it is likely that other factors were at play. The malaria policy was revised in 2005 and resulted in the introduction of ACTs for the treatment of uncomplicated malaria. Since ACTs were newly introduced, coverage of ACTs were likely very low in 2006; however only the latest DHS includes ACTs in its coverage estimates, which was reported as 15% in 2012. For the purposes of comparability between survey years however, only overall appropriate antimalarial treatment coverage was used for the coverage trend analysis. Other explanations for the drop in coverage may include frequent stock-outs of antimalarial drugs [19], however data on stock outs is only available from 2009. By June 2011, data retrieved from joint supervision activities pointed to an improvement in drug supply, including that of antimalarials. However, according to a 2013 census of facilities [34], large proportions of the drugs were expired, highlighting ongoing challenges in the country's supply

chain. Given the lack of supply data before 2009 however, it is hard to determine with certainty the impact the supply of anti-malarial drugs had on the declining coverage observed.

Despite serious challenges with food security, Niger has been able to achieve some reduction in stunting rates of children under-5. General improvements in stunting could be partly explained by reductions in the incidence and severity of infectious illnesses due to increased care-seeking as well as improvements in the proportion of children who are vaccinated. Our analysis reveals a four-fold increase in the effect of reduced childhood stunting on deaths averted. Yet those gains are virtually nullified by increases in wasting rates, reflecting a need to further invest in the country's resilience to recurrent droughts and address the structural causes of food insecurity. The country experienced two major food crises in the past decade; one in 2005 and the other in 2010. Increased severity of food shortages in the second of these droughts may account for the higher rates of wasting in 2012. Furthermore, with seasonal fluctuations affecting malnutrition rates, peaking between June to September [35], the 2012 DHS may have been more prone to reflect these variations due to its extension into June whereas the 2006 DHS was completed by May. While the impact of food insecurity and seasonal fluctuations on childhood nutrition is well known, it is important to bear in mind that such data fluctuations may also be a result of measurement and collection errors of these national demographic surveys.

By 2012, ASCs at health post level were responsible for approximately 15% of care provision for under-5 malaria, suspected pneumonia and diarrhoea; the increase in care-seeking at the health post level, while modest, is impressive given that iCCM services were only provided at scale by 2011. Routine data support this trend; in 2012 health posts provided 1.2 treatments per child per year or 24% of expected cases were treated at this level [36]. The expansion of health care access to the community through the construction of health posts, the deployment of ASCs and the removal of user-fees, has had an important impact on averting deaths in children in addition to other cost and convenience benefits of providing care closer to home [37]. In the absence of the ASCs, it is unlikely that higher level health centers and hospitals would have been able to meet the increasing demand for services. Our lives saved analysis demonstrates that care-seeking at community/health post level has resulted in an estimated 12% fewer deaths compared to a scenario where this level of care would not exist.

Our findings suggest that several factors worked synergistically to achieve the decreases in child mortality in Niger. Firstly, the introduction of new interventions, most notably the Hib vaccine introduced in 2009, which our LiST analysis shows contributed 14% to under-5 deaths averted. Secondly, from an economic perspective, the country has seen a steady increase between 2000 and 2008 (coinciding with the scale up of health infrastructure including the health posts) in per capita expenditure on health (in constant 2011 international dollars), rising from $33 per capita in 2000 to $60 in 2013 [16]. Furthermore, external resources for health saw a peak of around 30% of total expenditure on health in 2005/2006 (during the nutrition crisis) [38], but has fallen to around 12% since 2011 [16]. In December 2000, Niger qualified for enhanced debt relief from the International Monetary Fund (IMF) for Heavily Indebted Poor Countries (HIPC) [39] and in December 2005, the IMF announced that Niger received 100% multilateral debt relief, with approximately US $86 million debt being written off [40]. A subsequent agreement was reached for the 2012–2014 period for Niger to receive around $123 million under the IMF's Extended Credit facility [41]. The debt relief was key to enabling the Government of Niger to invest in its health system.

Of concern however is that out of pocket expenditure on health, which declined between 2000 and 2006 (from 65% to 51% of total expenditure on health [16]), increased again after 2006; this appears counterintuitive given the greater access to services during that period, although the free health care initiative was only made available to pregnant women and

children under-5. The increased access to services for all may have driven higher usage of services for individuals not covered under the free health care policy. By 2012, out of pocket expenditure had increased to 60% of total expenditure on health [16]; if this trend continues, it raises significant equity concerns.

Amouzou et al. [2] identified the lack of progress for newborn mortality as a key gap in Niger's child survival success, and our data confirm this. Reductions in newborn mortality are harder to achieve than under-5 mortality and will require concerted efforts by the government to expand access to maternal and newborn care. Our analysis should that while coverage of antenatal care increased, skilled care at birth has stagnated since 2006 and postnatal care coverage remains low at 17%.

Niger is increasingly facing a financial burden resulting from the free health care initiative. Annually, approximately US$7.8 million has been allocated to the free health care initiative, but this is estimated to be only half of what is needed. While government allocations began to improve by 2011, there remains a funding deficit [42]. With future government plans to replace ASCs with nurses to increase the spectrum of services provided in addition to up skilling and compensating the historically volunteer relais cadre to assume some of the current ASC responsibilities, health costs for the government will increase considerably. Donors provide substantial support to the iCCM initiative through the provision of free diagnostics and drugs, resulting in precarious sustainability of the country's health programmes. External support will therefore have to remain in the short to medium term.

This study draws on available household survey data, desk review, and key informant interviews and has aligned indicator definitions across the MICS, DHS and Mortality Survey to achieve a robust and comparable coverage trend analysis. The triangulation of quantitative data with a thorough desk review allowed for a multi-faceted analysis of the health programmes in Niger and for an interrogation of health coverage data within a broader contextual framework.

Data limitations should be kept in mind however. The various surveys are not strictly comparable, having used different sampling frames and being conducted at different times of the year resulting in expected seasonal variations in particular health indicators. While LiST was valuable in measuring the contribution of specific interventions to overall mortality reduction, as with all models it may be compromised by data of variable quality and there remains uncertainty around the intervention effectiveness values and coverage indicator inputs in LiST. Furthermore, due to the mix of methods used to collect contextual data, we were unable to quantify the relative contributions of wider changes in the health system and other distal determinants of health beyond facility and community based interventions to the reduction of child mortality. Accurate attribution of factors contributing to mortality change requires more information on the quality of preventive and clinical services. Our study did however follow the approach used in two previous country case studies [2, 43], one of which is part of the Countdown to 2015 multi-institutional, multi-agency collaboration to track progress towards MDG goals 4 and 5 [2]. This analysis will therefore add to the body of literature describing unique country pathways towards improved child survival.

## Conclusion

Niger has achieved significant reductions in child mortality which are plausibly due to the prioritization of primary health care services augmented by the establishment of a community-based platform through which iCCM could be delivered. The 2006 free health care policy and emphasis on primary health care was paramount in increasing the accessibility of health services to families, especially those most hard to reach. However the sustainability of this policy

and health service provision is in question. Niger's population is expected to nearly quadruple from 19 million in 2015 to 69 million by 2050 [44], placing immense pressure on the government to expand services in the face of unpredictable GDP growth. Furthermore, given the dependence on donor support for drugs and diagnostics and the increasing pressures on government funding, sustaining child survival gains will require ongoing external funding in the medium term.

## Ethical approval

This study was approved by the ethics committee of the South African Medical Research Council (EC026-9/2012). Approval was also provided by the UNICEF Niger country office. Data for the analysis of intervention coverage and mortality was taken from secondary sources (nationally representative household surveys) which are anonymized and de-identified prior to public release.

## Supporting Information

**S1 File.**
(DOCX)

## Acknowledgments

This evaluation was supported through Catalytic Initiative funding by the Department of Foreign Affairs, Trade and Development Canada (DFATD), the South African Medical Research Council and UNICEF. We thank the Niger Ministry of Health and UNICEF country office for their assistance with the field visit. TD and DS are supported by the National Research Foundation, South Africa. KK and MK are supported by Save the Children's Saving Newborn Lives program, which is funded by a grant from the Bill & Melinda Gates Foundation. We acknowledge the role of Dr David Hercot in the development of the protocol for this evaluation. We would also like to acknowledge the comments received from Dr Theresa Diaz, Prof Debra Jackson and Mr Christian Salazar on the manuscript draft which helped to strengthen the paper.

## Author Contributions

Conceived and designed the experiments: DB KK DS ED JR SR MK NL TD. Performed the experiments: DB DS ED KK NL SM. Analyzed the data: DB KK SM SR. Contributed reagents/materials/analysis tools: KK SM. Wrote the paper: DB KK NL DS ED SR JR WvD MK SM NPO FH AO AYG TD.

## References

1. UNICEF. Levels and Trends in Child Mortality: Report 2015. New York: UNICEF, 2015.
2. Amouzou A, Habi O, Bensaid K. Reduction in child mortality in Niger: a Countdown to 2015 country case study. Lancet. 2012. Epub 2012/09/25. doi: 10.1016/S0140-6736(12)61376-2 PMID: 22999428.
3. Canadian International Development Agency. The Catalytic Initiative to Save a Million Lives 2007. Available from: http://www.acdi-cida.gc.ca/acdi-cida/acdi-cida.nsf/eng/NAD-1249841-JLG.
4. UNICEF. UNICEF Final Report to CIDA 2007–2013. New York: UNICEF, 2014.
5. Leon N, Sanders D, Van Damme W, Besada D, Daviaud E, Oliphant NP, et al. The role of 'hidden' community volunteers in community-based health service delivery platforms: examples from sub-Saharan Africa. Global health action. 2015; 8:27214. doi: 10.3402/gha.v8.27214 PMID: 25770090.
6. Wang H, Liddell CA, Coates MM, Mooney MD, Levitz CE, Schumacher AE, et al. Global, regional, and national levels of neonatal, infant, and under-5 mortality during 1990–2013: a systematic analysis for the Global Burden of Disease Study 2013. Lancet. 2014; 384(9947):957–79. Epub 2014/05/07. doi: 10.1016/s0140-6736(14)60497-9 PMID: 24797572; PubMed Central PMCID: PMCPmc4165626.

7. UNICEF. At a glance: Niger 2013 [cited 2014]. Available from: http://www.unicef.org/infobycountry/niger_statistics.html.
8. Index Mundi. Niger Population growth rate 2014 [cited 2014]. Available from: http://www.indexmundi.com/niger/population_growth_rate.html.
9. UNHCR. UNHCR addresses needs of 20,000 forced to flee violence in Mali 2012. Available from: http://www.unhcr.org/4f312a219.html.
10. United Nations High Comissioner for Refugees. 2014 UNHCR country operations profile-Niger 2014 [cited 2014]. Available from: http://www.unhcr.org/pages/49e484ee6.html.
11. Thurston A. An Update on the Flood of Refugees from Libya into Niger 2011 [cited 2014]. Available from: http://sahelblog.wordpress.com/2011/05/20/an-update-on-the-flood-of-refugees-from-libya-into-niger/.
12. Flynn D, de Clercq G. Special Report: Areva and Niger's uranium fight: Reuters; 2014 [cited 2014]. Available from: http://www.reuters.com/article/2014/02/05/us-niger-areva-specialreport-idUSBREA140AA20140205?feedType=RSS&feedName = businessNews.
13. Storo C. Food insecurity in Niger: The potential for conflict and regional destabalisatio: Consultancy Africa Intelligence; 2010 [cited 2014]. Available from: http://www.consultancyafrica.com/index.php?option = com_content&view = article&id=480:food-insecurity-in-niger-the-potential-for-conflict-and-regional-destabilisation&catid=60:conflict-terrorism-discussion-papers&Itemid=265.
14. Bureau of African Affairs US Department of State. U.S. Relations With Niger: Fact sheet 2013. Available from: http://www.state.gov/r/pa/ei/bgn/5474.htm.
15. Trading Economics. Rural population (% of total population) in Niger: Trading Economics; 2015. Available from: http://www.tradingeconomics.com/niger/rural-population-percent-of-total-population-wb-data.html.
16. World Bank. World Development Indicators 2015 [December 2015]. Available from: http://search.worldbank.org/data?qterm = per%20capita%20GDP&language=EN
17. The World Data Bank. Health expenditure, public (% of government expenditure): World Bank Group,; 2014 [cited 2014]. Available from: http://data.worldbank.org/indicator/SH.XPD.PUBL.GX.ZS.
18. The Global Fund: a bleak future ahead. Lancet. 2010; 376(9749):1274. Epub 2010/10/19. doi: 10.1016/s0140-6736(10)61897-1 PMID: 20951874.
19. Leon N, Besada D, Daviaud E, Sanders D. Report on the Summative External Evaluation of the Catalytic Initiative (CI)/ Integrated HealthSystems Strengthening (IHSS) Programme in Niger. Cape Town, South Africa: South African Medical Research Council, 2014.
20. The World Data Bank. Out-of-pocket health expenditure (% of private expenditure on health): World Bank Group,; 2014 [cited 2014]. Available from: http://data.worldbank.org/indicator/SH.XPD.OOPC.ZS.
21. Republique du Niger, UNICEF. Enquête a Indicateurs Multiples de la fin de la Décennie: MICS2 Niamey: Ministry of Health and UNICEF, 2000.
22. Institut National de la Statistique Ministére de l'Èconomie et des Finances, Macro International Inc. Enquête Démographique et de Santé et à Indicateurs Multiples 2006. Niamey: Institut National de la Statistique, 2007.
23. Institut National de la Statistique, Ministére de l'Èconomie et des Finances. Enquete Nationale sur la Survie des Enfants de 0 a 59 mois et la mortalite au Niger 2010. Niamey: Institut National de la Statistique, 2011.
24. Institut National de la Statistique Ministére de l'Èconomie et des Finances, Macro International Inc. Enquête Démographique et de Santé et à Indicateurs Multiples 2012. Niamey: Institut National de la Statistique, 2013.
25. Pedersen J, Liu J. Child mortality estimation: appropriate time periods for child mortality estimates from full birth histories. PLoS Med. 2012; 9(8):e1001289. doi: 10.1371/journal.pmed.1001289 PMID: 22952435; PubMed Central PMCID: PMC3429388.
26. Rutstein S, Rojas G. Guide to DHS Statistics. Calverton: ORC Macro, 2006.
27. Bryce J, Terreri N, Victora CG, Mason E, Daelmans B, Bhutta ZA, et al. Countdown to 2015: tracking intervention coverage for child survival. The Lancet. 2006; 368(9541):1067–76.
28. Boschi-Pinto C, Black RE. Development and use of the Lives Saved Tool: a model to estimate the impact of scaling up proven interventions on maternal, neonatal and child mortality. International journal of epidemiology. 2011; 40(2):520–1. Epub 2010/12/22.: doi: 10.1093/ije/dyq171 PMID: 21172931.
29. Stover J, McKinnon R, Winfrey B. Spectrum: a model platform for linking maternal and child survival interventions with AIDS, family planning and demographic projections. International journal of

epidemiology. 2010; 39 Suppl 1:i7–10. Epub 2010/04/02. doi: 10.1093/ije/dyq016 PMID: 20348129; PubMed Central PMCID: PMC2845855.

30. Walker N, Tam Y, Friberg IK. Overview of the Lives Saved Tool (LiST). BMC Public Health. 2013; 13 (Suppl 3):S1. doi: 10.1186/1471-2458-13-S3-S1 PMID: 24564438

31. Friberg IK, Walker N. Using the Lives Saved Tool as part of evaluations of community case management programs. Journal of global health. 2014; 4(2):020412. doi: 10.7189/jogh.04.020412 PMC4267103. PMID: 25520802

32. Liu L, Oza S, Hogan D, Perin J, Rudan I, Lawn JE, et al. Global, regional, and national causes of child mortality in 2000–13, with projections to inform post-2015 priorities: an updated systematic analysis. The Lancet. 2014.

33. Central Intelligence Agency. Population Below Poverty Line 2014. Available from: https://www.cia.gov/library/publications/the-world-factbook/fields/2046.html.

34. Doherty T, Besada D, Zembe W, Daniels K, Kinney M, Kerber K, et al. Report on the summative external evaluation of the Integrated Health System Strengthening programme in Ethiopia, Mali, Mozambique, Ghana, Malawi and Niger. Cape Town: South African Medical Research Council, 2014.

35. Loutan L, Lamotte J. Seasonal variations in nutrition in Niger. Lancet. 1984; 1(8883):945–7.

36. Oliphant NP, Muniz M, Guenther T, Diaz T, Lainez YB, Counihan H, et al. Multi-country analysis of routine data from integrated community case management (iCCM) programs in sub-Saharan Africa. Journal of global health. 2014; 4(2):020408. doi: 10.7189/jogh.04.020408 PMID: 25520798; PubMed Central PMCID: PMC4267092.

37. Guenther T, Sadruddin S, Chimuna T, Sichamba B, Yeboah-Antwi K, Diakite B, et al. Beyond distance: an approach to measure effective access to case management for sick children in Africa. The American journal of tropical medicine and hygiene. 2012; 87(5 Suppl):77–84. doi: 10.4269/ajtmh.2012.11-0747 PMID: 23136281

38. Cornia GA, Deotti L. Niger's 2005 food crisis: Extent, causes and nutritional impact. Florence: University of Florence, 2008.

39. Niger To Receive US$890 Million In Debt Service Relief: The IMF and World Bank Support Debt Relief for Niger Under the Enhanced HIPC Initiative [Internet]. Washington, DC: International Monetary Fund,; 2000. Available from: https://www.imf.org/external/np/sec/pr/2000/pr0076.htm

40. International Monetary Fund. Niger: Fifth Review Under the Three-Year Arrangement Under the Poverty Reduction and Growth Facility and Requests for Waiver, Modification of Performance Criteria, and Extension of the Arrangement. Washington, D.C.: International Monetary Fund,, Department A; 2007.

41. Statement by IMF Managing Director Christine Lagarde at the Conclusion of her Visit to Niger [Internet]. 2011. Available from: https://www.imf.org/external/np/sec/pr/2011/pr11483.htm

42. Olivier de Sardan JP, LASDEL. Conférence nationale pour le renforcement de la gratuité des soins au Niger. Les retards de remboursement: Le problème, ses causes, ses conséquences. Joint Communities of Practice Workshop Bujumbura2012.

43. Masanja H, de Savigny D, Smithson P, Schellenberg J, John T, Mbuya C, et al. Child survival gains in Tanzania: analysis of data from demographic and health surveys. Lancet. 2008; 371(9620):1276–83. doi: 10.1016/S0140-6736(08)60562-0 PMID: 18406862.

44. UNICEF. Generation 2030 Africa. New York: UNICEF, 2014.
